# Biodegradable UV-Protective Composite Film from Cellulosic Waste: Utilisation of Cotton Gin Motes as Biocomponent

**DOI:** 10.3390/polym16010088

**Published:** 2023-12-27

**Authors:** Zengxiao Cai, Abu Naser Md Ahsanul Haque, Renuka Dhandapani, Maryam Naebe

**Affiliations:** 1 Institute for Frontier Materials, Deakin University, Geelong, VIC 3216, Australiaa.haque@deakin.edu.au (A.N.M.A.H.); 2 Cotton Incorporated, 6399 Weston Parkway, Cary, NC 27513, USA

**Keywords:** cotton waste, cotton ginning, single-use plastics, UV protection, biodegradable packaging

## Abstract

With an increase in environmental pollution and microplastic problems, it is more urgent now to replace non-biodegradable films with biodegradable films that are low-cost and from renewable resources. Cotton gin motes (GM), a type of cellulosic waste that is generated from cotton ginning, is an excellent candidate for fabricating biodegradable films due to its properties and abundance. In this study, GM was first mechanically milled into a fine powder, followed by compounding with polycaprolactone (PCL) and extruded to produce composite pellets which were then compress-moulded into composite films. This environmentally friendly process used physical processing and all the materials were consumed in the process without generating any waste residue. To improve the compatibility and mixing properties between GM and PCL, the use of a plasticiser (polyethylene glycol) was considered. A high content of GM powder (up to 50%) was successfully compounded with the polymer. The SEM images of the composite films showed smooth surface morphology and well-distributed GM powder in the PCL matrix. The added advantage of compounding GM with the polymer matrix was that the composite film developed UV-shielding properties due to the presence of lignin in the GM powder. This property will be critical for films used in UV-resistance applications. Furthermore, the composite even with high GM content (50%), showed good mechanical properties, with 9.5 MPa yield strength and 442% elongation, which was only a 50% decrease in elongation when compared with clear PCL film. The soil biodegradation of GM composite films under controlled temperature (20 °C) and humidity (50%) for 1 month showed around 41% weight loss. Overall, this study demonstrates the potential of GM to be used as a biodegradable and UV-protective composite film for a wide array of applications, such as packaging and UV-protective coverings.

## 1. Introduction

Cellulose is the most prevalent naturally occurring organic compound as at least one-third of all the plant matter on the earth is made of cellulose. The cellulose chains are composed tightly via different inter and intra-molecular hydrogen bonds [1]. Cellulose is a low-cost renewable resource with very high tensile strength (150 GPa) and modulus (10 GPa); hence, it is highly attractive as a reinforcing material for producing green composites [2].

Several forms of cellulose have been used in composites including nanocellulose [3], micronised particles [4], fibrous structures and regenerated cellulose [5]. Although longer cellulose fibres may have better mechanical performance, handling the longer fibres is difficult due to feeding issues and agglomeration, leading to poor dispersion [4]. Using the nanocellulose form (nanocrystals, nanofibres or bacterial cellulose) can be beneficial due to the high surface area, crystallinity [6], tensile modulus and complex viscosity [7], but as it is commonly fabricated during wet processing stages, the final product tends to agglomerate during drying and is hard to disperse in the polymer matrix during melt extrusion. Despite the benefits of nanocellulose in combination with different polymers being widely reported in the literature [8,9], poor dispersion characteristics of nanocellulose on a large scale have limited the industrial production of cellulose nanocomposites [4]. In this context, the use of micronised cellulose particles creates a balance between using either longer cellulosic fibres or nano-sized cellulose for extrusion-based composite preparation because of its simple preparation process and good dispersibility in the polymer matrix.

The sources of cellulose for composite preparation can be selected from a wide variety of options due to its abundance in nature. However, utilising cellulose from a waste stream is more beneficial in terms of minimising cost and having a positive environmental impact [10]. Cotton gin motes (GM) is a great resource of cellulose waste which is generated in cotton processing facilities across the world. This is a mixture of non-spinnable pure cotton fibres with some broken or immature seeds separated during the cotton ginning process. It has been reported that around 200,000 tons of GM are produced in the United States (USA) alone in a year [11], whereas the USA contributes just around 11.7% of the global cotton production [12]. GM contains around 67% cellulose, ~4% hemicellulose and ~13% lignin [13]. Interestingly, the cellulose content of GM is higher than that of many common natural resources such as eucalyptus (44.9%), pine (45.6%), spruce (47.1%), cedar (52.7%), and other plants including hemp (54%), and bamboo (46.5%) [14]. Due to these factors, using GM as a source of cellulosic material for diverse applications seems appropriate.

There is also an ongoing interest in using cotton ginning waste, such as gin trash in composite preparations with different polymers including polypropylene [15], polyvinyl alcohol [16] and polycaprolactone (PCL) [17]. However, GM requires separate attention in the composite preparation due to its significantly higher cellulose content than gin trash (30% cellulose) [13]. There have been some recent studies conducted on GM to extract nanocellulose, i.e., nanocrystals [18] and nanofibres [11], for various applications in line with the benefits listed.

Given that cellulose is a renewable resource that has influential properties, e.g., high tensile strength and modulus, it benefits the final composite products when compounded correctly with a polymer [19,20]. Since GM is also a resource with high cellulose content, it is important to identify the impact of GM in a composite structure. In this regard, another significant parameter of GM to note is its low hemicellulose content (4%). In a typical biomass structure, where cellulose, hemicellulose, and lignin are present, hemicellulose is known as the most unstable part with more hydrophilic properties than the other parts [21]. It is proven that the removal of hemicellulose can reduce the overall hydrophilicity [22] and improve the compatibility of biomass (such as wood) with hydrophobic polymers for composite preparation [21]. Therefore, compared to other common natural resources, GM is likely to be more compatible with hydrophobic polymers without the need for an extraction. In addition, due to the improved interfacial compatibility, it could be possible to reinforce a high amount of GM in the composites. This would be a sensible utilisation of GM. However, to date, there has been no study on using GM exclusively in an extrusion-based composite. Hence, exploring such an opportunity for GM is the highlight of this study. To achieve that, three objectives were considered: (1) preparation of the GM powder using mechanical process, (2) preparation of composite using best possible proportion of GM and (3) identifying the physicochemical properties and biodegradation trend of the composite. 

To prepare the composite, GM was first converted into a micro-sized powder since micronised cellulose powder has been proven as the best reinforcing form of cellulose for large-scale use [4]. Polycaprolactone (PCL) was selected as the polymer matrix in this study due to its proven success in extrusion with cellulosic materials, good flexibility and biodegradable nature [23,24]. The final form of the composite selected was a film, considering the potential application of this fully bio-based material in the widespread field of packaging, as a sustainable replacement to current non-biodegradable single-use plastics. 

## 2. Materials and Methods

### 2.1. Materials

**Figure 1:**
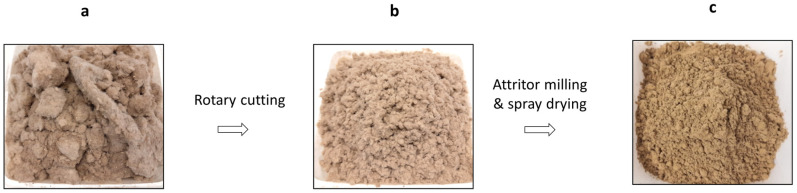
Conversion of (**a**) cotton gin motes (GM) into (**b**) coarse powder and (**c**) fine powder.

GM was sourced from New South Wales, Australia, during the cotton cropping season in 2022. The powder from GM was prepared following the method used in our previous study [25]. At first, GM was cut using a Pulverisette 19 rotary cutter mill (Fritsch GmbH, Idar-Oberstein, Germany). Then, the obtained coarse powder was milled in an attritor mill for 4 h (S/1, Union Process, Akron, OH, USA). After that, the prepared slurry was spray-dried (B-290, Buchi Labortechnik AG, Flawil, Switzerland) to obtain the final powder. Powders obtained after the cutter mill and attritor mill were named coarse powder and fine powder, respectively. Figure 1 shows digital images of GM and the powders at each stage. Polycaprolactone (PCL, CAPA 6800, Mw 80,000) was sourced from Era Polymers, Banksmeadow, Australia, and polyethylene glycol (PEG) of molecular weight (Mw) 10,000 was sourced from ChemSupply, Gillman, Australia. 

### 2.2. Fabricating GM/(PEG/PCL) Pellets

PEG flakes were ground into a powder form by using a variable speed rotor mill, i.e., ring grinder (Pulverisette 14, Fritsch, Idar-Oberstein, Germany), and then compounded with PCL at 90/10 and 95/5 PCL/PEG ratios and to achieve PCL/PEG pellets. The highest content of GM powder (4 h attritor milled) that could be compounded in the GM/(PEG/PCL) composite was 50%. In this study, both GM-50 and GM-40 pellets were produced for the preparation of composite films. The calculated actual percentage of each component used in the composite is summarised in Table 1.

### 2.3. Fabricating Films by Compression Moulding

The pellets were first spread evenly in a mould made of aluminium. The mould was then put inside a hot press setup (McMillan Engineering Group, Dandenong South, Australia) and maintained at 80 °C for 20 min to let the pellets melt. After that, the press (6.3 t) was applied at the same temperature (80 °C) for another 20 min. Then, the temperature was gradually increased step by step, i.e., 10 min at 90 °C, 10 min at 100 °C and 10 min at 105 °C. Following a similar method, a pure PCL film was also prepared as the control.

### 2.4. Characterisations

#### 2.4.1. Measurement of Particle Size

The particle size of GM slurries after 1 h, 2 h, 3 h and 4 h of attritor milling were determined in a particle size analyser (Mastersizer 3000, Malvern Instruments Ltd., Malvern, UK). The machine measured the size of particles with three consecutive scans and provided the mean values, which were reported. As per the extracted data, volume-based particle size distributions of the GM particles were plotted using Origin 2020.

#### 2.4.2. Morphology

The morphology of GM powder and the films (surface and cross-section) were observed using a Supra 55VP scanning electron microscope (SEM) facility (Zeiss, Oberkochen, Germany) using 5 kV accelerating voltage. All the samples were gold-coated using an EM ACE600 sputter coater (Leica, Macquarie Park, Australia). 

#### 2.4.3. Chemical Structure

The chemical structure of the samples was identified via Fourier-Transform Infrared (FTIR) spectroscopy in an attenuated total reflectance mode. The GM powder, PCL film, PEG flakes, GM-50 and GM-40 composite films were tested in a Vertex 70 (Bruker, Karlsruhe South, Germany) spectrometer. The parameters of testing were 4 cm^−1^ scan resolution, with 4000 cm^−1^–500 cm^−1^ wavenumbers and 64 scans for each sample. The data were extracted after a baseline correction. 

#### 2.4.4. Optical Transmittance

The optical transmission properties of the PCL film, GM-50 and GM-40 composite films were measured using a Cary 5000 Scan UV-Vis-NIR spectrophotometer (Varian Inc., Palo Alto, CA, USA) in the visible (400–800 nm) and UV wavelength region (200–400 nm). 

#### 2.4.5. Thermal Degradation

The thermal degradation behaviour of GM powder, PCL film, PEG flakes, GM-50 and GM-40 films were assessed in a thermal analyser (TGA Q50, TA Instruments, New Castle, PA, USA), using a temperature range from 30 °C to 500 °C and 10 °C min^−1^ heating rate. To obtain the Derivative Thermogravimetric (DTG) data, the temperature data were plotted on the x-axis, and derivative weight change (%) per degree of temperature was plotted on the y-axis. 

#### 2.4.6. Mechanical Properties 

The modulus of elasticity, tensile strength and elongation at the break of all the film samples were measured using an Instron 5967 universal tensile testing system (Instron, Norwood, MA, USA). For this purpose, a 100 N load cell was used with a 50 mm/min constant rate for elongation. The tensile properties were acquired in both yield and break. An average of data from three specimens for each sample was considered as the final result. 

#### 2.4.7. Biodegradation

The films were cut into rectangle shapes and some were buried in garden soil mix (Garden Basics, Mittagong, NSW, Australia) and some were left on top of the soil. In both cases, the soil was kept in containers and the containers were placed inside an incubator maintaining temperature at 20 °C and humidity at 50% (cold and dry conditions) for 30 days. Three specimens of each sample were kept in the incubator at the beginning and one from each set was taken out every 10 days, washed, dried and weighed. This procedure was triplicated to obtain average and standard deviation values for each sample at each time point. The dried biodegraded samples were also observed using a SEM technique to confirm the physical changes. 

### 2.5. Statistical Analysis

Statistical analysis of the results from mechanical tests and biodegradation was conducted using a two-tailed *t*-test, and probability (p) was calculated. Data were defined as statistically significant when p was found less or equal to 0.05.

## 3. Results

### 3.1. Particle Size and Powder Morphology 

**Figure 2:**
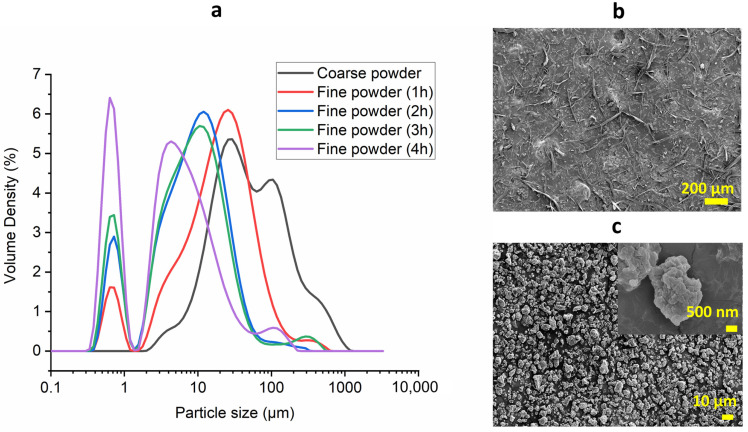
Particle size distribution of GM powder (**a**) at different stages of milling and powder morphology through SEM (**b**,**c**).

Figure 1a shows the particle size allocation of the GM powders at the different stages of milling. GM coarse powder showed two major powder size distributions, probably due to the presence of two distinct natures of the particles, one coming from the cotton fibres and one from the wooden fractions in GM. This was also consequenced in the GM slurries from 1 h to 4 h milling as two normal size distributions were seen. Table 2 shows the particle size of GM powders achieved after rotary cutting and attritor milling (1 to 4 h). The values of d(10), d(50) and d(90) represent the sizes of 10%, 50% and 90% of the powders that were lower than the listed size, respectively. The first hour of attritor milling reduced the d(50) by around 57.4% (from approx. 46 μm to 20 μm) via the continuous beating of the GM powder using the ceramic balls during the process. With the increase in attrition time, the d(50) reached 4.37 μm after 4 h. The time of attrition was optimised at 4 h as further attrition time resulted in minimum changes in the particle size [25]. The benefit of having a longer duration of attrition is usually to enhance the uniformity of particle shape. The morphology of GM coarse powder (Figure 1b) showed a mixture of fibres and randomly shaped particles related to the contents in GM. Powders showed non-spherical random shapes after the 4 h attrition, as shown in Figure 2c, due to the further breakdown of the GM coarse powders into a much smaller size.

### 3.2. Physical Appearance and Film Morphology

**Figure 3:**
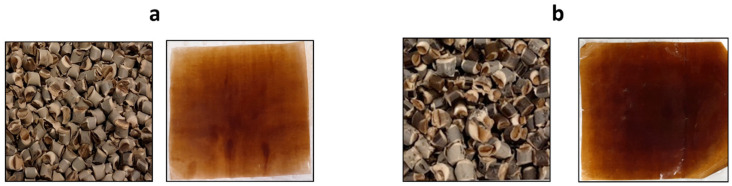
Composite pellets and films of (**a**) GM-40 and (**b**) GM-50.

GM composite pellets and films are shown in Figure 3. The different percentages of plasticiser (PEG) and GM were examined to reach the best and highest portion of GM in the composite. GM at 40% and 50% were the highest concentrations that could be successfully extruded into smooth and continuous filament. These filaments were then chopped into uniform-sized composite pellets as shown in Figure 3. The brown colour of composite pellets and films was due to the partial presence of lignin in GM. The GM composite films were measured to be 105 ± 10 µm thick.

**Figure 4:**
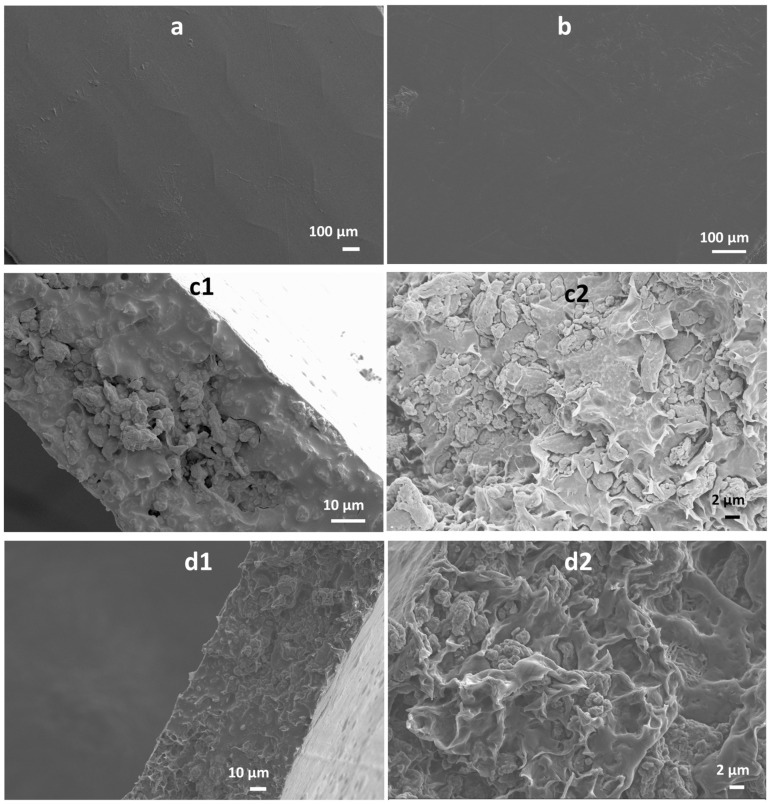
Scanning electron microscopic (SEM) images of the surface of (**a**) GM-50 and (**b**) GM-40 film, and cross-section of (**c**) GM-50 and (**d**) GM-40 at (1) lower and (2) higher magnifications.

The surface and cross-section morphologies of GM composite films are shown in Figure 4. The surface of the GM composite films was relatively smooth as observed in Figure 4a,c. From the cross-section morphologies, even from the higher magnification images, it is also evident that GM powders were mixed well with PCL and distributed evenly in the film (Figure 4b,d). The well-distributed GM powder in the composite films could be attributed to the micronised size of the particles used in the composite matrix. This can be confirmed from different studies where the micronisation of cellulosic fillers by ball milling was found to be beneficial in terms of achieving good distribution [4], improved filler-matrix interaction, good melt processability and better mechanical properties [26]. 

Apart from that, PEG as a plasticiser could also have an impact by occupying the intermolecular spaces in the cellulose chain and interrupting the intermolecular forces of cellulose [16], thereby reducing the structure rigidity and making it more compatible with the PCL interfaces. 

### 3.3. Chemical Structure

**Figure 5:**
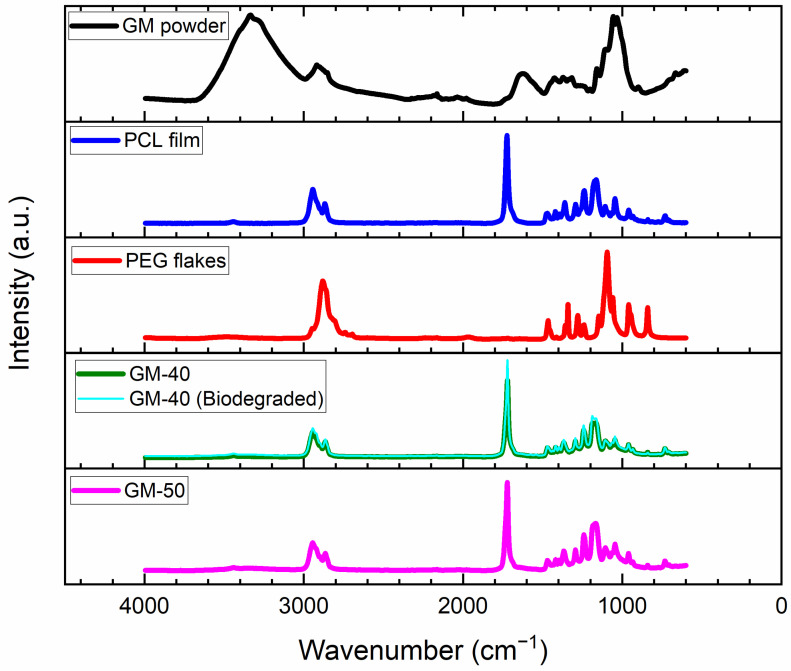
FTIR spectra of GM powder, PCL film, PEG flakes, GM-50 and GM-40 films, and GM-40 film after biodegradation.

The FTIR spectra of GM powder, PCL film, PEG flakes and GM composite films are shown in Figure 5. The O–H stretching vibration of around 3338 cm^−1^ corresponded to the lignocellulose structure in GM powder, which shifted to 3445 cm^−1^ for GM-50 and GM-40 composite films, possibly due to the establishment of hydrogen bonding between GM with the polymers, i.e., PCL and PEG [17]. The symmetric and asymmetric CH_2_ stretching were seen in all the spectra at 2868 cm^−1^ and 2943 cm^−1^, respectively. The carbonyl stretching of PCL was seen at around 1726 cm^−1^ [27] and in composite films, though it was not present in the cellulosic structure of GM. The C=C stretching was seen at around 1620 cm^−1^ due to the presence of lignin in GM powder, though it was not observed in the composite films. However, the peak at 1375 cm^−1^ due to the phenolic OH group in lignin was seen in GM and the composite samples. The alcoholic C–O stretching was seen near 1240 cm^−1^ [17] in all the spectra of PEG, PCL and GM composite films. The C–O–C ether in PEG and PCL showed peaks around 1095 cm^−1^ and 1175 cm^−1^, respectively [28]. The peaks between 1028 and 1060 cm^−1^ were related to the peaks from C–O–C stretching vibrations in cellulose structure and existed in both the GM powder and GM composite films [16]. There was no difference found in the chemical characteristics of samples before and after biodegradation as the spectra (for GM-40) overlapped each other. Overall, the FTIR results of the composite samples were found as a combination of the respective chemical groups of each component. These spectra were reproducible in different places on the films, suggesting a well-balanced dispersion of the three components through the melting process. 

### 3.4. Optical Transmittance

**Figure 6:**
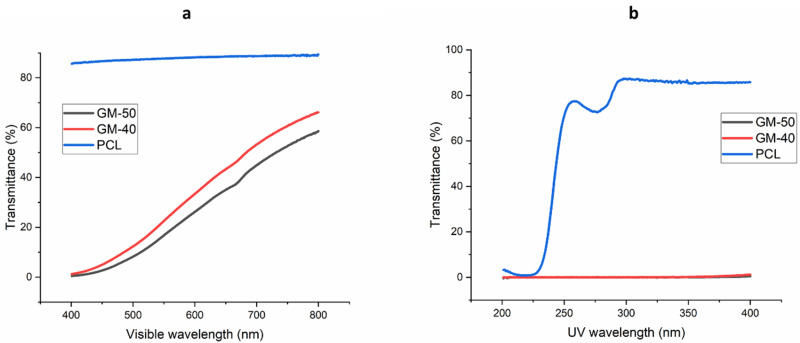
Transmission spectra of PCL, GM-50 and GM-40 composite films in (**a**) visible wavelength and (**b**) UV wavelength.

The light transmittance of PCL film, GM-50 and GM-40 composite films under visible and UV lights are shown in Figure 6a and Figure 6b, respectively. As expected, the pure PCL sample demonstrated a high transparency in the visible light region (greater than 80%). Its transmission at 200 nm was 2.3% and a band near 275 nm to 400 nm indicated its absorption of UV light. On the other hand, for the developed bio-based plastic films, the visible light transmission decreased from 800 nm to 450 nm. The GM-50 composite film showed lower light transmission compared with the GM-40 composite film due to its darker colour (Figure 3). The darker colour in the film was due to the presence of more GM fraction that contained relatively higher lignin content. This was proved by the band near 680 nm for both the bio-based composite films. This band was due to the presence of lignin in GM, which contains different chromophore groups, e.g., guaiacyl (for yellow-brown colour), syringyl (for red-purple colour) and aromatic rings [17]. The zero UV transmission of GM composite films from 400 nm to 200 nm was related to the presence of lignin in GM. The UV resistance property in the plastic film has previously been reported to be beneficial in varied applications, such as food packaging, to prevent nutrient degradation and mulching by inhibiting weed growth [17]. 

### 3.5. Thermal Degradation

**Figure 7:**
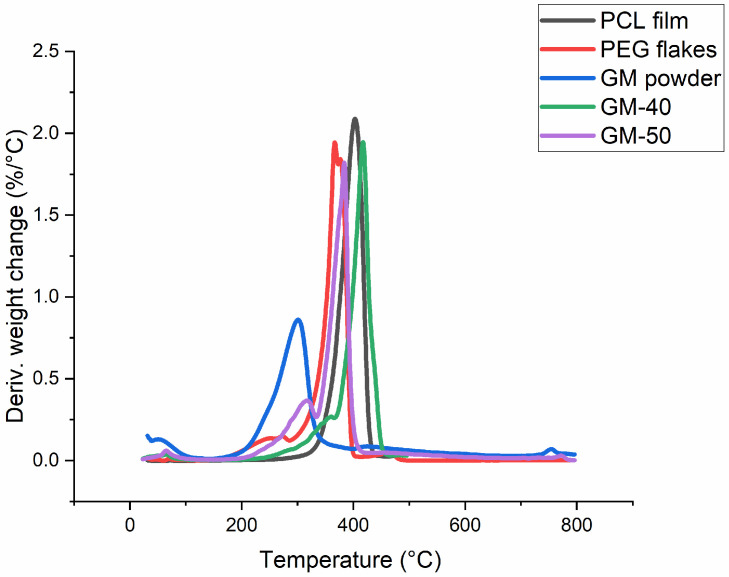
Derivative Thermogravimetric (DTG) behaviour of GM powder, PCL film, PEG flakes, GM-50 and GM-40 composite films.

The DTG curves for GM powder, PCL film, PEG flakes, GM-50 and GM-40 composite films are shown in Figure 7. The temperatures at which 10% and 50% of weight loss occurred in each sample are also listed in Table 3. For GM powder, loss of weight from 30 °C to 100 °C was mainly from moisture evaporation, whereas the degradation of powder started around 210 °C as its main content was cellulose [29]. The degradation of PEG started near 183 °C and a 97% loss of weight occurred at 399 °C, whereas the degradation of PCL started near 290 °C and a 97% loss of weight was seen at 442 °C. None of the polymers existed at 500 °C due to complete degradation. The GM-50 composite film started degradation from nearly 214 °C and lost 50% weight at 370 °C, though it maintained nearly 10% weight from 463 °C to 500 °C. The GM-40 composite film started degradation from near 224 °C and lost 50% weight at 410 °C, but it maintained a 10% weight from 444 °C. Both the composites maintained a similar weight (~10%) at 500 °C, indicating that the difference in GM content was not significant enough to influence the overall thermal stability. Both the composite films did not completely degrade at 500 °C, which aligns with the data of pure GM (20% weight maintained at 500 °C) and is associated with the higher thermal stability of lignin [1].

### 3.6. Mechanical Property

The modulus of elasticity, tensile strength, and elongation of the samples at yield and break are presented in Table 4. Overall, the modulus of elasticity of GM-50 film (345.5 MPa) and GM-40 film (465.1 MPa) was found to be higher than that of PCL film (275.4 MPa); however, their tensile strength and elongation at both yield and break were observed lower than that of pure PCL film. At the breaking point, GM-50 film showed a decrease of around 50.9% elongation when compared to PCL. The elongation at yield and breaking point for GM-50 film was 4.6% and 441.5%, respectively, which were lower than those of GM-40 (6.5% at yield and 477.7% at break). This could be related to the higher cellulosic fraction in the GM-50 sample, as cellulose is known for its high modulus and low elongation properties [16,17]. 

Due to the PCL chain’s natural extensibility, the breaking strength of PCL was much higher. However, after a certain point, due to the necking behaviour where a substantial reduction occurs in the cross-section, calculating the breaking strength will be meaningless. It is unclear how necking will affect the gauge length as a whole before the break, but according to ASTM standards, the values after the yield of this kind of curve only have a qualitative purpose [30]. Therefore, when comparing the yield strength as a reference, the GM-50 film showed a 28.4% higher strength than that of the GM-40 film. The yield strength of GM-50 composite film showed a 35% decrease when compared to pure PCL film. 

Even though the strength and elongation were found to be decreased, this is not unexpected when a large amount of cellulosic filler is incorporated into the PCL matrix. For example, a 50% reinforcement of bacterial cellulose in the PCL matrix has been reported to reduce 98.2% of tensile strength, and the elongation dropped by 98.9% in comparison with PCL film [31]. In another study, 30% amorphised cellulose reinforced in PCL showed a 68.5% decrease in elongation at break compared with PCL [32]. A higher amount of filler often produces more voids and roughness which impacts the mechanical properties of the composite. However, the results in the current study suggest that higher filler content (50%) of GM particles in the PCL matrix results in an acceptable reduction in mechanical strength. The morphology of the films as seen in the SEM suggested that even with a high amount of filler, the distribution of particles was still uniform. The strength of the films formed was also in the range of many commercial plastics, such as Teflon (2–5.6 MPa), polyethylene (PE) (2.5–13.8 MPa) and polyvinyl chloride (PVC) (2.3–11.5 MPa) [33]. 

### 3.7. Biodegradation

**Figure 8:**
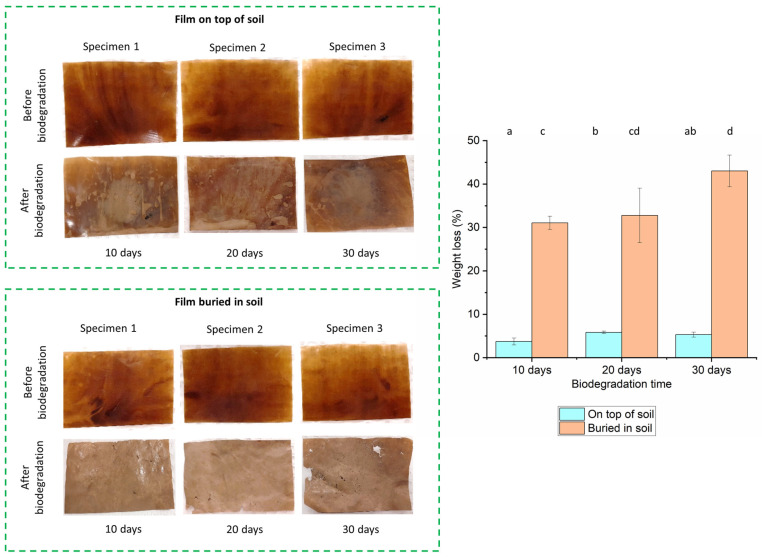
The physical changes in GM-40 composite films before and after soil degradation and weight loss (%) during biodegradation. Different alphabets indicate data that are significantly different (*p* ≤ 0.05) from each other.

**Figure 9:**
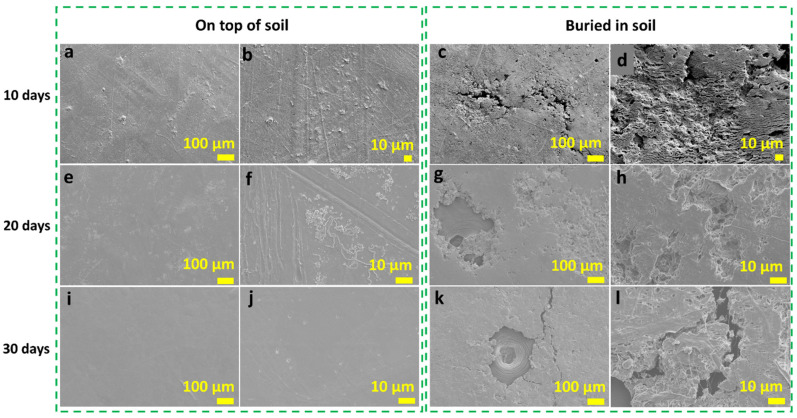
SEM images of GM-40 composite film surface after biodegradation (on top of the soil and while buried in soil): (**a**–**d**) after 10 days, (**e**–**h**) after 20 days and (**i**–**l**) after 30 days.

The physical changes from biodegradation for 30 days in the GM-40 composite films both on top and buried in soil, along with the weight loss calculated during this period, are shown in Figure 8. Here, GM composite films with 3% PEG content were investigated for biodegradation as a higher amount of PEG (i.e., 5%) has already shown a good biodegradation rate in our previous cotton gin trash composite film study (68% weight loss after 30 days) [17]. As seen in Figure 8, for the samples on top of the soil, the colour faded in some parts of the film during the biodegradation period. The films maintained almost the same appearance as their original films after 10 days (~3.8% weight loss), 20 days (~5.6% weight loss) and 30 days (~5.3% weight loss) of biodegradation. This can be further confirmed by their SEM morphology in Figure 9 with only a few small pores observed on the surface. However, films buried in the soil lost their initial colour with rough and porous surfaces observed (~31% weight loss) after 10 days. For the film buried in the soil for 20 days, the colour became lighter, with a consistently rougher surface and missing parts on the edge (~32.7% weight loss). The statistical analysis suggested that for the samples degraded on top of the soil, there was no specific trend, and there was no significant difference between samples after 10 days and 30 days. This indicates that the samples were much more stable on top of the soil for the whole period. However, for the samples buried in the soil, despite the differences between the 10-day and 20-day samples, and between the 20-day and 30-day samples which were not significant, the difference between the 10-day and 30-day samples was statistically significant (*p* ≤ 0.05). This indicates the significant gradual loss in the samples’ weight over time, which aligns with the increasing average weight loss percentage values with the progression of time. More pores were also revealed in the SEM image on the surface of the samples (Figure 9g,h). After 30 days of biodegradation, the GM-40 composite film lost around 41.1% of its weight with its initial appearance and structural integrity undergoing significant changes with large pores and cracks formed on the surface, as perceived from the digital image in Figure 8 and SEM images in Figure 9k,l. The higher amount of weight loss results for the buried films compared to the films on top of the soil achieved in this study suggests a substantial level of biodegradation of the GM-40 composite films by the microorganisms present in the soil [34]. 

## 4. Conclusions

In this study, GM composite film was first successfully produced using a sustainable fabrication process without any chemical treatment or generation of any waste residue. GM powder was prepared using a complete mechanical process achieving a particle size of 4.37 µm. As a sustainable cellulosic filler, a high content of micro-sized GM powder (up to 50%) was compounded in the GM/PCL composite matrix, while morphology observations revealed good distribution of the powder in the matrix and a smooth surface on the final composite film. The developed films completely blocked UV light (200–400 nm) due to the presence of lignin in GM. The addition of a high amount of filler did not negatively impact the tensile strength (12 MPa) and elongation (442%) of the composite films, which fell in the range of commercial single-use plastics. The FTIR analysis showed no chemical alterations of cellulose or PCL in the composite before or after the biodegradation. The composite films were thermally more stable compared to the control PCL due to the presence of lignin. The composite sample showed quicker biodegradation when buried in the soil (around 40% weight loss) compared to when placed on top of soil (around 5% weight loss). Based on the lack of UV transmission and the biodegradation results obtained, it is clear that the newly developed GM composite film will be a promising replacement for non-biodegradable plastic films for numerous applications, such as packaging, UV-protection covering and mulching.

## Figures and Tables

**Table 1:** The nomenclature of the combinations used and the percentage of each component.

Name	Components (%)		
	GM	PEG	PCL
GM-50	50	5	45
GM-40	40	3	57

**Table 2:** The size particles in GM coarse powder and attritor-milled powders.

Milling	Time (h)	d(10) μm	d(50) μm	d(90) μm
Cutter milling	-	12.2	46.2	241
Attritor milling	1	3.19	19.7	65.3
	2	0.89	8.65	27.4
	3	0.764	7.81	27.3
	4	0.595	4.37	20.7

**Table 3:** Weight loss phenomena of the samples from thermal analysis.

	Temperature (°C)	
	10% Weight Loss	50% Weight Loss
GM powder	216	303
PCL film	369	403
PEG flakes	273	363
GM-40 film	336	410
GM-50 film	284	370

**Table 4:** Mechanical properties of PCL, GM-50 and GM-40 films. Different superscripts in the same column indicate data that are significantly different (*p* ≤ 0.05).

	Modulus (MPa)	Elongation (%)		Tensile Strength (MPa)	
		Yield	Break	Yield	Break
PCL film	275.4 ± 6.0 ^a^	12.9 ± 1.4 ^a^	900.8 ± 88.7 ^a^	14.7 ± 1.5 ^a^	40.2 ± 6.0 ^a^
GM-40	465.1 ± 22.4 ^b^	6.5 ± 1.7 ^b^	477.7 ± 121.4 ^b^	7.4 ± 0.1 ^b^	9.3 ± 1.1 ^b^
GM-50	345.5 ± 56.1 ^a^	4.6 ± 2.0 ^b^	441.5 ± 49.7 ^b^	9.5 ± 0.4 ^c^	12.1 ± 0.8 ^c^

## Data Availability

The data presented in this study are available upon request from the corresponding author.
